# Investigating the burden of mental distress among nurses at a provincial COVID-19 referral hospital in Indonesia: a cross-sectional study

**DOI:** 10.1186/s12912-021-00596-1

**Published:** 2021-05-12

**Authors:** Marthoenis, Maskur, Liza Fathiariani, Jacqueline Nassimbwa

**Affiliations:** 1 Universitas Syiah Kuala, Banda Aceh, Indonesia; 2 Zainoel Abidin General Hospital, Banda Aceh, Indonesia; 3 Aceh Psychiatric Hospital, Banda Aceh, Indonesia; 4 Health Plus Development Communications (HeDCO), Makerere off Bativa Road, P.O.Box 880, Kampala, Uganda

**Keywords:** Prevalence, Financial, Depression, Anxiety, Stress, Nurse, COVID-19

## Abstract

**Background:**

The current outbreak of the COVID-19 pandemic has distorted the physical, mental, and psychological condition of frontline healthcare providers in health facilities. This study aims to investigate the prevalence, and risk factors of depression, anxiety, and stress among nurses working in a COVID-19 referral hospital in Indonesia.

**Methods:**

A cross-sectional study was conducted among 491 nurses, aged between 31, and 56 years, using a self-administered questionnaire. The Depression Anxiety Stress Scale (DASS21), and demographic questions were used to screen the presence of psychological problems, and their associated factors.

**Results:**

The prevalence of moderate to extremely severe depression, anxiety and stress was 8.5 %, 20.6 and 6.3 %, respectively. Regression analysis showed that anxiety was significantly higher among nurses working in non-COVID wards (*p* = .01), those who experienced social rejection (*p* < .05), and those who frequently watched television (*p* < .05). Those who had temporary contracts were more stressed (*p* < .05), and those who faced financial hardship during the COVID-19 pandemic significantly experienced depression, anxiety, and stress at 10.5 %, 23.5 %, and 8.1 % respectively(*p* < .05).

**Conclusions:**

While the prevalence of mental distress in this hospital is low, it exists, and hospital management should consider training for all nurses, public sensitization on COVID-19, and provision of financial subsidies for frontline workers, in order to manage the risk factors.

## Background

The corona virus disease (COVID-19) has caused substantial health burdens globally [1]. With increased unpredictability of the evolution of the extremely communicable virus and consequent uncertainty of the end of this pandemic [2], many countries instituted preventive protocols, and measures to control the spread. These included mandatory isolations in form of lockdown of all social- economic activities to minimize face to face interaction [2, 3]. The change in environment created by pandemics is known to create varied manifestations of mental distress such as anxiety, fear, depression, frustration, anger, loneliness, and stress, among others. For example, in Malaysia, the halting of economic activities translated into financial struggles. Institutions resorted to staff and salary cuts, as well as unpaid leave [4]. Employees who have to experience such consequence develop a sense of insecurity and uncertainty which translate into mental distress.

A Systematic Review by Talevi et al. (2021) shows that the beginning of the COVID-19 pandemic (March 28, 2019 – April 3, 2020) created anxiety, depression and post traumatic symptoms globally. Most people experienced mild-moderate symptoms, and a few showed severe symptoms of mental distress. The review further shows that the most affected persons are frontline health workers and COVID-19 patients [3].

Increased mental health burdens among front line health workers is related to the higher risk of infection and disease. These carders also worry about infecting their family members, and they carry a burden of watching their patients die [4, 5]. Earlier studies reveal poor sleep quality [6], depression, anxiety, stress [7, 8], Post Traumatic Stress Disorder and burnout following the influx of hospitals with COVID-19 patients [9]. A Systematic Review and Meta-analysis of 93 studies published between January and September 2020 [10] shows that approximately one-third of nurses working during the COVID-19 pandemic suffered from psychological symptoms. The study specifically discovered a pooled prevalence rate of 35 %, 37 and 43 % for depression, anxiety and stress, respectively.

Mental distress among nurses has been linked with various risk factors and comorbidities. Depression, for example is positively associated with female gender, being single, having children, living with a person aged 60 years or older [11], lower education level, working in a critical care unit [12]. In addition, it is negatively associated with self-efficacy, resilience, as well as intra and extra-family social support [5]. Additionally, the anxiety of nurses is positively associated with female gender, being single, having workload increased [11], working in a critical care unit [12], being suspected with COVID-19 infection, insufficient personal protective equipment [13] and negatively with higher self-efficacy, resilience, intra and extra-family social support [5]. Stress on the other hand has also been positively associated with female gender and age ≤ 30 years, as well as watching or reading COVID-19 news ≥ two hours per day, poor family and social support [14], fear of infection, and fear transmission to the family member. Furthermore, it has been negatively associated with having training courses on COVID-19, availability of personal protective equipment and obtaining special attention from hospital administration [15].

Psychological intervention for those at higher risk of common mental health problems should be an integral part of work plans suggested in fighting the outbreak [16]. Team cohesion, as well as government and social support have been reported to improve mental health among frontline health workers in pandemics [5, 17]. The wide range of risk factors and comorbidities of nurses which increase their burden of common mental health problems necessitates special attention, prevention and intervention.

The paucity of related evidence from Indonesia was a predisposition for this study which aimed to assess the burden of depression, anxiety and stress, as well as investigate if socio-demographic factors have an effect on these mental distress variables among nurses working at the emergence of the COVID-19 pandemic in Indonesia.

## Methods

### Study design and Participants

A cross-sectional online study was conducted from 20th July to 19th August 2020 among all nurses working in a provincial COVID-19 referral hospital in Indonesia. The nurses in this hospital are employed under one of two contract terms - for a fixed term (usually one year) defined by the hospital (temporary nurse) or permanently by the government (permanent nurse). At the onset of the outbreak in early 2020, the hospital assigned several nurses, regardless of their employment status to a one-day training on COVID-19. These were thereafter assigned to work in newly created wards for COVID-19 patients. All 745 nurses providing care (to all patients regardless of their COVID-19 status) in the hospital were included in this study. Those nurses that occupy administrative positions were excluded because they do not directly attend to patients. In this paper, the term “COVID-19 nurse” is used to refer to those nurses who directly manage patients with COVID-19, while regular nurses are those working in regular (non-COVID-19) wards.

Using a contact list for all nurses working in the hospital, each nurse was invited to participate in the study via a private WhatsApp message. A total of 745 messages were sent to all included nurses by a senior nurse (MR) who is part of the research team and an author on this article. Of these, 140 nurses were assigned to COVID-19 wards, while 604 nurses worked in the regular wards. The message had an introduction and explanation about the study, as well as a link to a google form with pre-designed questions. A maximum of three follow ups were made with each nurse through WhatsApp messaging and phone calls. A total of 491 nurses responded to the invitation by participating in the study, 258 did not respond.

### Measures

A Google Form was designed to collect socio-demographic information on age, gender, latest education, employment status, duration of employment, and marital status. The form also included questions on personal finances during the COVID-19 pandemic, as well as experience of social rejection because they worked in a COVID-19 hospital. Furthermore, the presence of depression, anxiety and stress was measured using the Depression Anxiety Stress Scale (DASS-21). This scale has 7 criteria for each of the variables (depression, anxiety and stress), totaling to 21 criteria for self-reporting characteristic attitudes and symptoms [18]. Each criterion is scored on a 4-point Likert scale (0 = did not apply to me at all, to 3 = applied to me very much, or most of the time. However, the original DASS had 42 criteria and this was shortened to 21 to make it easy for respondents. Before interpreting the scores therefore, the scores were multiplied by two in order to obtain a similar comparison with the original DASS-42 [19, 20]. The DASS-21 also has been consistently used to study the presence of depression, anxiety and stress, not only among nurse, but also other healthcare workers and general population [10, 20–24].

For depression scale, the scores of 9 or less, 10–13, 14–20, 21–27 and 28 or greater were considered “normal”, “mild”, “moderate”, “severe” and “extremely severe”, respectively. For anxiety scale, scores of 7 or less, 8–9, 10–14, 15–19 and 20 or greater were considered “normal”, “mild”, “moderate”, “severe” and “extremely severe”, respectively. In addition, for stress scale, scores of 14 or less, 15–18, 19–25, 26–33 and 34 or greater were considered “normal”, “mild”, “moderate”, “severe” and “extremely severe”, respectively [18].

The DASS-21 is an easy-to-use screening instrument which is reliable and has been well utilized globally. Furthermore, it has been translated into various languages and has high reliability [25–28]. In this study, the overall reliability coefficient (Cronbach alpha) of DASS-21 scale used was 0.94, while for each sub-scale was 0.84, 0.86 and 0.86 for depression, anxiety and stress, respectively.

### Statistical analysis

Descriptive statistics for socio-demographic variables including gender, age group, marital status and education were generated. The normal distribution of numerical data and the correlation between numerical variables were tested using the Shapiro-Wilk and Spearman correlation tests, respectively. In order to test the association between the socio-demographic and the presence of moderate to extremely severe mental distress, a chi-squared test was used. The Shapiro Wilk test showed that the data was not normally distributed and for this reason, a Mann Whitney U test was used to examine the association between socio-demographic variables and the DASS-21 scores. Multiple logistic analysis was performed to detect factors associated with the presence of depression, anxiety and stress. Data analysis was carried out using the STATA 13 software [29].

## Results

### Socio - Demographics

Of the 491 nurses who completed the online questionnaires (response rate = 65.9 %), 79.8 % (*n* = 392) worked in regular wards, and 20.2 % (*n* = 99) worked in COVID-19 wards. Most participating nurses were female 72.5 % (*n* = 375), and most were married 75.4 % (*n* = 370). As many as 75.6 % (*n* = 371) had been working in the hospital for 10 years or less while 61.7 % were temporary nurses. Also, 55 % (*n* = 303) were aged between 31 and 56 years, and 57.8 % (*n* = 284) had a nursing diploma as the highest level of education. Table 1 shows the different demographic characteristics between regular and COVID-19 nurses.

**Table 1:** Difference of demographic and mental problem between COVID-19 nurses and regular nurses (*n* = 491)

No	Characteristics	Total n (%)	COVID-19 Nurses, n (%)	Regular Nurses, n (%)	x2	*P*-value
1	Gender					
	Male	135 (27.5)	45 (45.5)	90 (22.9)	20.1	0.001
	Female	356 (72.5)	54 (54.5)	302 (77.1)		
2	Age group					
	22–30	221 (45)	53 (53.5)	168 (42.8)	3.64	0.056
	31–56	270 (55)	46 (46.5)	224 (57.2)		
3	Education					
	Nursing Diploma	284 (57.8)	63 (63.6)	221 (56.4)	1.7	0.191
	Bachelor of Nursing	207 (42.2)	36 (36.4)	171 (43.6)		
4	Employment status					
	Temporary	303 (61.7)	75 (75.8)	228 (58.2)	10.35	0.001
	Permanent	188 (38.3)	24 (24.2)	164 (41.8)		
5	Employment duration					
	≤ 10 years	371 (75.6)	86 (86.8)	285 (72.7)	8.58	0.003
	≥ 11 years	120 (24.4)	13 (13.2)	107 (27.3)		
6	Marital Status					
	Unmarried	121 (24.6)	29 (29.3)	92 (23.5)	1.44	0.23
	Married	370 (75.4)	70 (70.7)	300 (76.5)		
7	Any financial problem during COVID-19pandemic					
	No	159 (32.4)	27 (27.2)	132 (33.7)	1.67	0.22
	Yes, getting worst	332 (67.6)	72 (72.7)	260 (66.3)		
8	Depression					
	Yes	42 (8.5)	10 (10.1)	32 (8.2)	0.37	0.53
	No	449 (91.5)	89 (89.9)	360 (91.8)		
9	Anxiety					
	Yes	101 (20.6)	14 (14.1)	87 (22.2)	3.13	0.07
	No	390 (79.4)	85 (85.8)	305 (77.8)		
10	Stress					
	Yes	31 (6.3)	5 (5.1)	26 (6.6)	0.33	0.56
	No	460 (93.7)	94 (94.9)	366 (93.4)		

### The prevalence of depression, anxiety and stress

As many as 20.6 % of the nurses experienced anxiety, followed by moderate to extremely severe depression (8.5 %) and stress (6.3 %), respectively. There was no significant difference found between results for COVID-19 nurses and the regular nurses on depression (*p* = .44) and stress (*p* = .52). However, the nurses in the regular (non-COVID-19) ward experienced significantly more anxiety symptoms (*p* = .01) than their peers in the COVID-19 wards. The nurses that experienced financial hardship due to the pandemic were significantly more affected, with 23.5 %, 10.5 and 8.1 % for anxiety, depression, and stress (*p* < .05), respectively. Further statistical analysis also confirms the poor association between other socio-demographic variables (age, education, employment status, employment duration, marital status) and the three mental distress variables (*p* > .05). Table 2 shows the details of the relationship between the DASS-21 scores and the socio-demographic characteristics.

**Table 2:** Relationship between DASS-21 score and nurses’ socio demographic characteristics

**Variable**	**Depression**	**Anxiety**			**Stress**	
Gender						
Male	U = -0.67	U = 1.43			U = 1.18	
Female	*p* = .49	*p* = .15			*p* = .23	
Age						
22–30	U = 0.34	U = 0.91			U = 0.30	
31–56	*p* = .73	*p* = .36			*P* = .75	
Education						
Nursing Diploma	U = 0.45	U = 0.23			U = 0.09	
Bachelor of Nursing	*p* = .65	*p* = .81			*p* = .92	
Employment status						
Temporary	U = -1.31	U = -1.25			U = -1.19	
Permanent	*p* = .18	*p* = .20			*p* = .23	
Employment duration						
≤ 10 years	U = 0.08	U = 0.96			U = 0.98	
≥ 11 years	*p* = .92	*p* = .33			*p* = .32	
Marital Status						
Unmarried	U = -0.08	U = 0.41			U = -0.034	
Married	*p* = .93	*p* = .67			*p* = .97	
Any financial hardship during COVID-19 pandemic						
No	U = -2.86	U = -2.8			U = -3.48	
Yes, getting worst	*p* = .004	*p* = .004			*p* = .001	
Working Place						
COVID-19 ward	U = 0.75	U = 2.37			U = 0.64	
Regular (Non COVID-19) ward	*p* = .44	*p* = .01			*p* = .52	
Have children						
No	U = -0.17	U = 0.17			U = -0.10	
Yes	*p* = .86	*p* = .85			*p* = .91	

### Associated risk factors of depression, anxiety and stress

A regression analysis showed that depression was associated with various factors. Firstly, optimism that the government may win against COVID-19 (AOR = 0.39; 95 % CI: 0.19–0.81) and appropriate behavior such as wearing a face mask whenever they leave their homes (AOR = 0.06; 95 % CI: 0.008–047) were independently associated with depression (*p* < .05). However, the nurses that experienced social rejection by the family as a result of their proximity to COVID-19 patients had approximately 3 times odds of being depressed, and those that felt rejected by the neighbors had 5 times odds. In addition, those that frequently watched the news on TV about COVID-19 had approximately 3 times odds of having depression.

Experiencing anxiety was also found to be associated with various factors, including going to crowded places such as the market, feeling worried about the pandemic and experienced rejection because of working in the hospital, either by family or neighbors. In addition, those who repeatedly watched news about COVID-19 on had higher odds of suffering from anxiety symptoms (*p* < .05). Meanwhile, the odds of suffering from anxiety were 64 % lower among the COVID-19 nursing team than the general nurses (AOR = 0.36; 95 % CI: 0.17–0.75). Temporary nurses also had almost 4 times odds of suffering from stress. Those who experienced rejection by the family because of working in the hospital had almost 6 times odds of suffering from the stress (*p* < .05). Table 3 shows the detail of multiple regression analysis for the risk factors of depression, anxiety and stress among nurses.

**Table 3:** Multiple logistic regression for risk factors of Depression, Anxiety and Stress among Nurses

**Variable**			**AOR**	**SE**		***P*-value**		**95 % CI**
Depression								
Sure, that the government can win against COVID-19			0.39	0.14		0.013		0.19–0.81
Wearing mask whenever going out from home			0.06	0.06		0.007		0.008–0.47
Rejection by the family because of working in the hospital			3.06	1.52		0.025		1.15–8.13
Rejection by the neighbours because of working in the hospital			5.05	2.4		0.001		1.99–12.83
Repeated and frequent news about COVID-19 in the TV made you anxious			2.98	1.4		0.02		1.18–7.50
Anxiety								
Being part of COVID-19 nursing team			0.36	0.13		0.006		0.17–0.75
Went to crowded places in last few days			1.88	0.45		0.008		1.17–3.02
Feeling worried about the COVID-19 pandemic			3.57	2.04		0.026		1.16–10.95
Rejection by the neighbours because of working in the hospital			5.43	1.78		0.001		2.85–10.33
Repeated and frequent news about COVID-19in the TV made you anxious			1.99	0.59		0.02		1.11–3.57
Stress								
Being a temporary staff of the hospital			3.86	2.13		0.01		1.31–11.38
Rejection by the family because of working in the hospital			5.97	2.52		0.001		2.60–13.66

Note: only statistically significant variables were included

## Discussion

This study presents the burden of mental distress experienced by the nurses at a COVID-19 referral hospital in Indonesia. 8.7 %, 5.8 and 20.6 % of the nurses, experienced depression, stress and anxiety respectively. However, these rates are lower than pooled rates reported in a recent meta-analysis, which are 43 %, 35 and 37 % for the same variables respectively [10]. Lower rates are however not unique to our study. Hong et al. 2020 found rates of 9.4 and 8.1 % for depressive and anxiety symptoms respectively, among nurses in China [30]; Salopek-Ziha et al. (2020) found 11 %, 17 and 10 % rates of depression, anxiety and stress, respectively among Croatian nurses and physicians [31]; In Italy, the rate of depression, anxiety and stress, was 8 %, 9.8 and 8.9 % respectively [22]. In contrast, other studies found more than two-thirds of nurses or healthcare workers suffered from mental distress [10].

While, different settings, methods and tools used for data collection might explain the variation in rates of mental distress between studies, some of the reasons for the low rates in the studies include targeted training on COVID-19, adopting proper mental health strategies [32] having proper occupational protection practices and access to good Personal Protective Equipment (PPE) that meet the nursing work requirement [21]. Considering that similar interventions were implemented by the hospital management in our study, they may have contributed to the lower rates that we found. Nevertheless, there is mental distress and interventions may need to be strengthened.

While the general prevalence is low, we found different risk factors for mental distress. Firstly, of the three symptoms, anxiety was significantly higher among the regular (non- COVID-19) nurses than the those working in COVID-19 wards (*p* = .01). Having observed from studies mentioned above that training, and access to PPE, among others contribute to improved mental health, the lack of such intervention for the regular nurses may be a possible explanation for increased anxiety. They may have lacked adequate information about the disease and its prevention or may have had fears about contracting the disease from their colleagues in the COVID-19 wards. As such, hospital administrations may consider awareness raising or scaling training and other interventions among all health workers regardless of whether they work in COVID-19 or regular wards.

Our study also found a significantly higher rate of depression, anxiety and stress among nurses with financial hardship during the pandemic. Such struggle during this period has been widely reported, either in general population [33, 34], among students or adolescents [35] or patients with chronic illness [36, 37]. At the time of the study (July – August 2020), the countrywide lockdown in Indonesia was underway. This financial hardship may have been related to the halt in all socio-economic activity and resultant consequences such as reduced income to the hospital as a result of reduced patient visits, or increased pay cuts or increased costs of services such a transport and food leading to reduced disposable income. Considering that the proportion of nurses that expressed financial struggles during the pandemic was high (67.6 %, *n* = 332), other studies should further investigate this variable in more hospitals. Comparison studies should also be conducted on similar experiences among health workers in COVID-19 referral hospitals and non-COVID hospitals.

Social rejection by family and neighbors because of working in the COVID referral hospital is also a risk factor for depression and anxiety. Similar findings have been reported among health workers in disaster situations generally [5]. Considering that our study was conducted in the first few months of the pandemic, the rejection experienced by nurses may have been due limited public information on prevention, care and treatment and the probable fear of contracting the disease. Hu et al. (2020) posit that the symptoms of depression and anxiety among frontline nurses are lowered by intra and extra family social support [5]. As such therefore, increased public awareness of the disease, adherence to preventive behavioral protocols such as hand hygiene and use of face masks, social rejection of nurses in the COVID referral hospital may reduce, along with public fear. The introduction to the COVID vaccine may also reduce fear of contracting the disease and related rejection for health workers in COVID-19 referral hospitals.

Additionally, repeatedly watching COVID-19 related news on TV independently contributed to the presence of depression and anxiety. Similar findings were found in Egypt and Saudi Arabia among health workers who watched news related to the COVID-19 pandemic for more than two hours per day [14], as well as citizens in China who were exposed to social media, official media, commercial media and overseas media about the pandemic [38]. While this finding may provide an insight on the role of Indonesia’s media in communicating about pandemics, the cross-sectional design of this study may not imply a temporal relationship between watching COVID-19 related news and mental distress. A bigger study is appropriate to investigate the impact of COVID-19 news on the mental health of the larger Indonesian population.

We further found that a temporary nurse was 3.8 times more likely to exhibit stress than the permanent staff. A heavier workload among temporary nurses has been reported in earlier studies [39–41]. In our study setting, the work contracts for temporary nurses are renewed annually and these depend on work performance. These nurses also get lower payment and have limited career development opportunities. On the other hand, the permanent staff have long contracts which last until retirement, and as such, better and stable monthly salaries, higher incentives, career development opportunities and pension funds. The limited advantage bore by the temporary staff might be a possible reason for higher stress scores. Additionally, the desire to find a stable job and income, as well as the burden to maintain a good work performance may also inflict mental distress.

Our study also shows factors that are associated to reduced mental distress. These include being part of COVID-19 team and optimism that government may win the battle against COVID-19. Working in teams promotes mutual support among frontline workers. A qualitative study conducted among an Australian medical team and nurses who were in Wuhan to provide medical assistance at the height of the pandemic in January 2020, suggests that working as team provides mutual support. The authors use the term “comradeship” to refer to this team [17]. According to the Cambridge dictionary, this term is defined as, “the feeling of friendship between people who live or work together, especially in a difficult situation” [42]. In our study, the finding of being part of a team and reduced likelihood of anxiety may be explained by the sense of comradeship. A qualitative study would provide more insight on this finding.

The finding that relates optimism and positive mental health is not new [43]. A study among breast cancer patients shows that those that scored higher on optimism reported better mental and social wellbeing, compared to those that were pessimistic [44]. Optimistic people are likely to adhere to healthy lifestyles, may have greater flexibility and may be better problem solvers [45]. Relatedly, it is likely that the nurses in our study that were optimistic about government efforts are more likely to heed to healthy protective behavior, work more diligently and focus less on the negative effects of the disease. The finding however illustrates the central role of government is spreading messages of hope and demonstrating efforts to end the pandemic.

### Limitations

First, the cross-sectional design could not infer the causation between the independent and outcome variables. Secondly, the study was conducted in a general hospital, thus could not represent the nurse’s condition in other provinces or settings. Thirdly, the self-selection bias could also contribute to the limited generality of study results. Future studies therefore should consider using a longitudinal design, multiple settings and a higher response rate.

## Conclusions

The prevalence of mental distress, especially depression (8.5 %) and stress (6.3 %) found in this study population was low compared to other studies. Generally, however, the nurses that work at the frontline of the COVID pandemic still experience mental distress albeit at different levels. This study shows that higher prevalence for mental distress was found among those who worked in regular wards, those who had financial hardship, those who watched COVID-19 related news, those with temporary work contracts, and those who experienced social rejection. In order to manage the risk factors, hospital management should consider interventions such as training and mental health protective measures on COVID-19 for all frontline health workers and not only those working in the COVID-19 wards. Increased public sensitization on COVID-19 may reduce fear and increase social support for these frontline cadres, while consideration of financial subsidiary could buffer them from financial uncertainty during the pandemic.

## Data Availability

The datasets analyzed in this study are available from the corresponding author on request.

## Abbreviations

COVID-19: Coronavirus disease 2019
DASS-21: Depression Anxiety Stress Scale
PPE: Personal Protective Equipment
